# A combined algorithm for T-wave alternans qualitative detection and quantitative measurement

**DOI:** 10.1186/1749-8090-8-7

**Published:** 2013-01-14

**Authors:** XiangKui Wan, Kanghui Yan, Dehan Luo, Yanjun Zeng

**Affiliations:** 1 School of Information Engineering, Guangdong University of Technology, Guangzhou, 510006, China; 2 Biomedical Engineering Center, Beijing University of Technology, Beijing, 100022, China

**Keywords:** T-wave alternans, Continuous wavelet transform, Rank-sum test, Quantitive estimation

## Abstract

**Background:**

T-wave alternans (TWA) provides a noninvasive and clinically useful marker for the risk of sudden cardiac death (SCD). Current most widely used TWA detection algorithms work in two different domains: time and frequency. The disadvantage of the spectral analytical techniques is that they treat the alternans signal as a stationary wave with a constant amplitude and a phase. They cannot detect non-stationary characteristics of the signal. The temporal domain methods are sensitive to the alignment of the T-waves. In this study, we sought to develop a robust combined algorithm (CA) to assess T-wave alternans, which can qualitatively detect and quantitatively measure TWA in time domain.

**Methods:**

The T wave sequences were extracted and the total energy of each T wave within the specified time-frequency region was calculated. The rank-sum test was applied to the ranked energy sequences of T waves to detect TWA qualitatively. The ECG containing TWA was quantitatively analyzed with correlation method.

**Results:**

Simulation test result proved a mean sensitivity of 91.2% in detecting TWA, and for the SNR not less than 30 dB, the accuracy rate of detection achieved 100%. The clinical data experiment showed that the results from this method vs. spectral method had the correlation coefficients of 0.96.

**Conclusions:**

A novel TWA analysis algorithm utilizing the wavelet transform and correlation technique is presented in this paper. TWAs are not only correctly detected qualitatively in frequency domain by energy value of T waves, but the alternans frequency and amplitude in temporal domain are measured quantitatively.

## Background

Sudden cardiac death (SCD), frequently ascribed to sustained ventricular arrhythmias, is one of the leading causes of mortality. American Heart Association claims approximately 350,000 lives annually in the US (approximately one every 1.7 minutes). Accurate identification of patients at increased risk for sustained ventricular arrhythmias is critical for the development of effective strategies to prevent SCD.

Traditionally, left ventricular ejection fraction was used to identify high-risk individuals and to assess the utility of the prophylactic administration of antiarrhythmic agents [1]. However, this strategy has no survival benefit [2]. There is now evidence that implantation of an internal cardioverter-defibrillator (ICD) in patients who are yet to experience sustained ventricular arrhythmias can improve survival [3-5]. But the costs and risks of indiscriminate application of ICD therapy may be unacceptably high. Some of the non-invasive tests related to high-risk SCD include ventricular late potentials and QT dispersion. However, the positive predictive value of these tests is too low to consider them as sufficient to make a decision about specific treatment, especially defibrillator implantation. The challenge is to develop new selective non-invasive methods which will allow the identification of high-risk patients before a major arrhythmic event occurs.

Recently, the T-wave alterenans (TWA) has been considered as one of the most promising markers, which allows identification patients at an increased risk for ventricular arrhythmia [6-13]. TWA is a phenomenon appearing in the ECG as a consistent fluctuation in the repolarization morphology on an “every-other-beat” basis (2:1 behavior). This fluctuation refers to a beat-to-beat variability in the amplitude of the T wave or ST segment. Numerous clinical studies have demonstrated the link between these oscillations and ventricular arrhythmias.

Current most widely used TWA detection algorithms work in two different domains: time and frequency.

Energy Spectral Method published by Adam et al. in 1981 [7] is the first quantitative studies relating TWA with myocardial instability. After that many researchers have presented many different algorithms based on it, such as Spectral Method (SM) [8], Complex Demodulation [9], and Karhunen-Loève Transform [10]. The spectral analytical techniques permit the registration of the alternans along the T wave by analysis of the power spectrum for each sample point. The disadvantage of the methods is that they treat the alternans signal as a stationary wave with a constant amplitude and a phase, which is not true in general. They cannot detect non-stationary characteristics of the signal.

The typical time-domain methods include Modified Moving Average method [11] and Correlation Method [12]. Time domain methods has also been used on Holter data, and it can detect TWA in short-time, non-stationary electrocardiogram (ECG) signal. But the higher quality of ECG signal is required, and the reliability and robustness of the algorithms need be improved further.

Besides above reported methods, several nonlinear methods and statistical methods of detection TWA are also presented, such as Laplacian Likelihood Ratio Method, Statistical Tests Method, Poincaré Mapping Method. The use of the methods as an immediate predictor of adverse cardiac events has, as far as the authors are aware, not been reported to date.

The wavelet transform has emerged over recent years as a powerful time–frequency analysis and signal coding tool favored for the interrogation of complex nonstationary signals. The continuous wavelet transform (CWT) has been used successfully in the processing of ECG signals, and offers significant advantages—in particular the preservation of feature-specific locations [13]. And Inaki used a wavelet transform-based methodology to detect the TWA in ECG qualitatively [14].

### The continuous wavelet transform (CWT)

The continuous wavelet transform is a time–frequency analysis method. It differs from the traditional short time Fourier transform by allowing arbitrarily high localization in time of high frequency signal features. The CWT is able to decompose a signal into different frequency components and one can study each of them with a different resolution, and a large selection of localized waveforms can be employed as long as they satisfy predefined mathematical criteria (described below). The wavelet transform of a continuous time signal, *x(t)*, is defined as:

(1) $$T\left(a,b\right)=\frac{1}{\sqrt{a}}\int_{-\infty }^{+\infty }x\left(t\right)\Psi^{*}\left(\frac{t-b}{a}\right)\mathrm{dt}$$

where Ψ^*^(*t*) is the complex conjugate of the analysing wavelet function Ψ^*^(*t*), *α* is the dilationparameter of the wavelet and *b* is the location parameter of the wavelet.

The contribution to the signal energy at the specific *α* scale and *b* location is given by the two-dimensional wavelet energy density function known as the scalogram:

(2) $$E\left(a,b\right)={\left|T\left(a,b\right)\right|}^{2}$$

In this paper we concern with the CWT as it allows arbitrarily high resolution in the time–frequency plane that has been found especially useful in the analysis of complex biosignals, most notably the ECG [15].

## Methods

### ECG signal preprocess

Broadly speaking, ECG contaminants can be classified into the following categories:

● power line interference

● baseline wandering

● electrode pop or contact noise

● patient–electrode motion artifacts

The power line interference is narrow-band noise centered at 60 Hz (or 50 Hz) with a bandwidth of less than 1 Hz, and an aptive notch filter is used to remove it [16]. Baseline wandering is estimated with a third order spline fitted to successive TP intervals. The spline is then subtracted from the ECG. The other noise and artifacts are suppressed by wavelet-based denoise technique [17].

The R peaks are located using the modulus maximum pair of wavelet transform. The stability of the heart rate is tested (standard deviation of RR < 10% mean RR). This test was designed to exclude ECGs with large RR variations, since the morphology of the T wave may be affected by a sudden change in heart rate.

### Combined Algorithm (CA)

Wilcoxon Rank Sum test is a statistical method, which can be used to test the null hypothesis that two populations X and Y have the same continuous distribution. If the TWA exists in a piece of ECG data, which means the amplitudes of T wave are consistent fluctuation, then the energy spectrums of T waves, which can be calculated by formula (2), appear the same fluctuation. And the values obtained are separated into two groups, corresponding to odd and even T-waves. Obviously, the two groups can be considered as two independent samples with unknown distribution, which meets the requirement of Wilcoxon Rank Sum test. So the Wilcoxon rank sum test was considered appropriate for the statistical analysis to obtain the probability that the groups come from the same population.

According to reference [14], the qualitative detection algorithm is described as follows.

1) When R peaks are detected by modulus maximum pair of wavelet transform, the mean RR intervals are calculated. And the ECG time-frequency information can be obtained.

2) Take the R peak as a fiducial point, T wave onset is calculated by formula (3) [18]

(3) $$Ts_{k}=40+1.33\sqrt{RR_{k}}\;\mathrm{ms}$$

Where *k* is the *k* th T wave, *k* = 1, 2, ⋯. And the width of analyzed T wave window is 400 ms. A temporal interval of 400 ms from *Ts*_*k*_ is considered to delimitate the whole T-wave.

**Figure 1:**
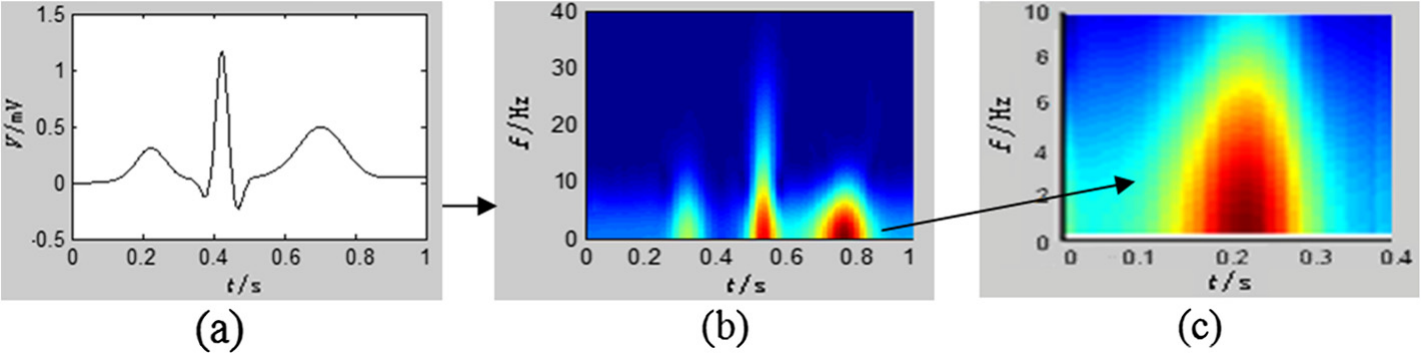
**Time-frequency feature extraction of T wave. **(**a**) a rhythm; (**b**) wavelet temporal-frequency figure of the rhythm (**c**) total energy of the T wave.

3) The frequency range of T wave is defined as being between 0.5 and 10 Hz.

4) For each delimited time–frequency region in the wavelet transform time–frequency plane, corresponding to the T wave, the total energy contained within the selected time–frequency region is calculated by formula (2) and extracted, which was shown as Figure 1.

5) All the energy values obtained are separated into two groups, corresponding to odd and even T waves. Then the Wilcoxon rank-sum test was applied to the two groups of values, and the probability that the groups come from the same population is then calculated. If the probability is below 0.05 (*P* < 0.05), that means the two groups are assumed to come from different populations and a TWA is deemed to have been detected. Otherwise, the signal is classified as exhibiting no TWA.

For the detected ECG signal existing TWA, A correlation technique is used to measure TWA quantificationally [12].

1) The median T wave (*T*_*m*_) is computed from the consecutive T waves of the detected ECG signal existing TWA.

(4) $$T_{m}=\sum_{i=1}^{N}T_{i}/N$$

Where N is the total number of T waves from the analyzed ECG signal.

2) An alternans correlation index (*ACI*_*i*_) is computed to measure morphological changes of each of the consecutive *T*_*j*_ waves in comparison to *T*_*m*_.

(5) $$\mathrm{AC}I_{i}=\frac{\sum_{j=1}^{M}T_{i}\left(j\right)T_{m}\left(j\right)}{\sum_{j=1}^{M}{\left[T_{m}\left(j\right)\right]}^{2}}$$

Where *M* is the number of sampling points of each T wave. The *ACI*_*i*_ indicates the alternans level of *T*_*j*_ in comparison to *T*_*m*_. If there exists at least 7 consecutive T waves whose *ACI*_*i*_ are alternating, then TWA start is considered in the first *T* wave of the consecutive T waves. And the TWA stop is considered in the last T wave of the consecutive T waves.

3) The TWA amplitude (*ACA*_*i*_) for *T*_*i*_ wave can be calculated using the formula (6):

(6) $$\mathrm{AC}A_{i}=2\left|\mathrm{AC}I_{i}-1\right|\frac{\sum_{j=1}^{M}T_{m}{\left(j\right)}^{2}}{\sum_{j=1}^{M}T_{m}\left(j\right)}$$

And the median TWA amplitude ($A\bar{C}A$) for the analyzed data is calculated by the formula (7)

(7) $$\overset{―}{\mathrm{ACA}}=\frac{\sum_{i=1}^{N}\mathrm{AC}A_{i}}{N}$$

Since Correlation technique tracked T waves in time, it was able to detect short-time T wave amplitude changes, as well as the number and the length of alternating segments in the series of beats, and the number percent of alternating T waves, which means it can quantificationally analysis the TWA in time domain.

### Simulated data

In actual ECG recordings, the exact value and timing of the TWA episodes are unknown. And such signals cannot be used to test algorithms. For that reason artificial ECG with added synthetic TWA are used to test the algorithms in this paper.

The artificial ECG model is defined as formula (8).

(8) S=e+k·a+l·w

Where S is the artificial ECG, *e* is the clean ECG signal obtained as the periodic repetition of a single beat, which guarantees that all the T waves are identical and so no TWA can be present in the original signal. *a* is the TWA episode, *k* is the alternans level, *w* is the noise, and *l* is the mixed noise factor. For the artificial ECG, four different noise sources have been considered: simulated Gaussian white noise, and three records of physiological noise from the MIT-BIH Noise Stress Test database: baseline wandering, muscular activity; electrode motion.

The specific obtained process of artificial ECG (i.e., S) is described as below.

1) A healthy subject underwent 10-min ECG recording sampled at 500 Hz and quantified with 5 μV/LSB in resting conditions, and a heartbeat, duration of 1 second, is selected. The clean ECG is formed by periodic repetition of a single beat 1000 times. The other 4 clean ECG segments from other 4 healthy subjects are formed in the same way. Then 5 clean ECG segments are obtained.

2) The shape of Gaussian function and its first order derivative (half of the whole waveform) are used as the alternans waveform separately. And there are five different alternans level for each alternans waveform, i.e. 10, 20, 50, 100, 200 μV. So 10 different TWA can be obtained, which are added the every T wave of above 5 clean ECG segments. And 50 ECG segments containing TWA are synthesized.

3) After linear superposition of above mentioned four different noises, the mixed noise are added to the artificial 50 ECG segments containing TWA. By adjusting the mixed noise factor *l* to different level, Signal-to-noise ratios are 20, 25, 30, 35 and 40 dB. And finally 250 noised ECG segments containing TWA (*S* = *e* + *k* · *a* + *l* · *w*) are obtained.

### Clinical data

The sudden cardiac death holter database and the European ST-T database are used as the clinical data sources. These databases are chosen by two reasons: firstly, previous studies found T-wave alternans episodes, some of them related to annotated ischemic episodes. Secondly, the databases are well-known and available by many research groups.

In the specific, a group of twenty five patients, who survived an acute myocardial infarction were considered. Each subject underwent 30-min ECG recording in resting conditions. And ECG segments were randomly extracted.

### Performance assessment

The detector performance needs to be evaluated with regard to the detection rate duration and magnitude of detected episodes. The validation of the detector should begin with a comparison of the simulated TWA episodes and the detector output in terms of sensitivity (*S*_*e*_). The sensitivity is defined as the number of correctly detected episodes divided by the total number of simulated episodes, i.e.

(9) $$S_{e}=\frac{\mathrm{TP}}{\mathrm{TP}+\mathrm{FN}}\times 100\%$$

Where TP is the number of true positive, i.e., the number of correctly detected ECG segments containing TWA. FN is the number of false negative, i.e., the number of missed ECG segments containing TWA.

## Results

### Simulated data test

The TWA detection algorithm described in the previous section was tested with the 250 artificial ECG test set.

The algorithm classified as TWA (*p <* 0.05) 228 (91.20%) of the overall simulated ECGs, i.e., the number of true positives was 228 and the number of false negatives was 22. the Sensitivity of the algorithm was 91.2%. the detection results under different SNR and alternans levels were shown in Table 1.

**Table 1:** Qualitative detection experiment data

**SNR/dB**	**P < 0.05**	**Se/%**	**TWA(μV)**	**P < 0.05**	**Se/%**
20	31	62.0	10	37	74
25	47	94.0	20	42	84
30	50	100	50	49	98
35	50	100	100	50	100
40	50	100	200	50	100
total	228	91.2		228	91.2

We can find from the Table 1 that the algorithm classified (i.e., *p <* 0.05) 100.00% of the signals containing TWA when the SNR is above 30. Alternatively, considering now the effect of the amplitude of the artificial TWA added to the signal, the algorithm classified as containing TWA 100.00% of the artificial TWA with amplitudes of 100 μV, 98% with 50 μV.

We also find statistically that the algorithm classified 92.0% of the signals with no added noise as containing TWA under different SNR (SNR = 20, 25, 30, 35, 40).

Further, the quantitative measurement of TWA was implemented to above qualitative detected ECG segments. The results were shown in Table 2.

**Table 2:** quantitative measurement TWA (unit: μV)

**Simulated TWA**	**Max alternans amplitudes**	**Min alternans amplitudes**	**Mean**$A\bar{C}A$
10	8.01	7.21	7.53
20	15.95	14.67	15.35
50	38.30	37.02	37.59
100	76.19	74.12	75.21
200	150.71	149.09	150.04

Given the simulated TWA amplitude, the relative error (RE) of TWA measurement is defined as follows

(10) $$\mathrm{RE}=\left(A_{\mathrm{twa}}-\bar{A}\right)/A_{\mathrm{twa}}$$

Where *A*_*twa*_ is the simulated TWA amplitude, and $\bar{A}$ is the measured TWA amplitude. *RE* represented the deviation percent of $\bar{A}$ from *A*_*twa*_.

The combined algorithm and SM gave the detection result for the same simulated ECG segment with 30db SNR in Table 3. (ASM: the measured TWA amplitude by SM).

**Table 3:** TWA detection results of CA and SM

**TWA/μV**	$A\bar{C}A$	**ASM**	**RE**_**ACA**_**/%**	**RE**_**ASM**_**/%**
10	7.5	3.4	25	66
20	15	6.9	25	65.5
50	37.8	17.4	24.4	65.2
100	75.2	34.8	24.8	65.2
200	152	69.6	24	65.2

We can found that the CA got the smaller RE compared with SM. $A\overset{―}{C}A$ was greater than ASM (220%), and less than simulated TWA(75%), and the measured TWA value was closer true TWA value.

### Clinical data test

The algorithm was used for the analysis of real ECGs selected from the above mentioned clinical database.

1-min lengths of ECG were considered that gave between 60 and 80 beats for normal sinus rhythm. And 30 segments of the 1-min length ECG signal were extracted from the selected patients, the sudden cardiac death holter database and the European ST-T database separately. They were analyzed using the algorithm described in Section III and SM separately. And the Partial results are shown in Table 4.

**Table 4:** Test results of partial samples

**Number**	**Data source**	***P*< 0.05**	$A\bar{C}A$**/μV**	**ASM/μV**
ECG1	Subject 1	11(37%)	4.92	2.41
ECG2	Subject 2	7(23%)	3.21	1. 23
31	SCDHD	19(63%)	18.72	9.09
36	SCDHD	10(33%)	10.12	4.61
41	SCDHD	23(77%)	5.12	2.39
45	SCDHD	25(83%)	14.96	7.10
46	SCDHD	9(30%)	9.12	4.71
e0119	ST-TD	17(56%)	16.78	7.31
e0121	ST-TD	22(73%)	21.82	9.83
e0125	ST-TD	12(40%)	17.85	8.11
e0601	ST-TD	0	0	0
e1302	ST-TD	10(33%)	7.12	3.45

**Figure 2:**
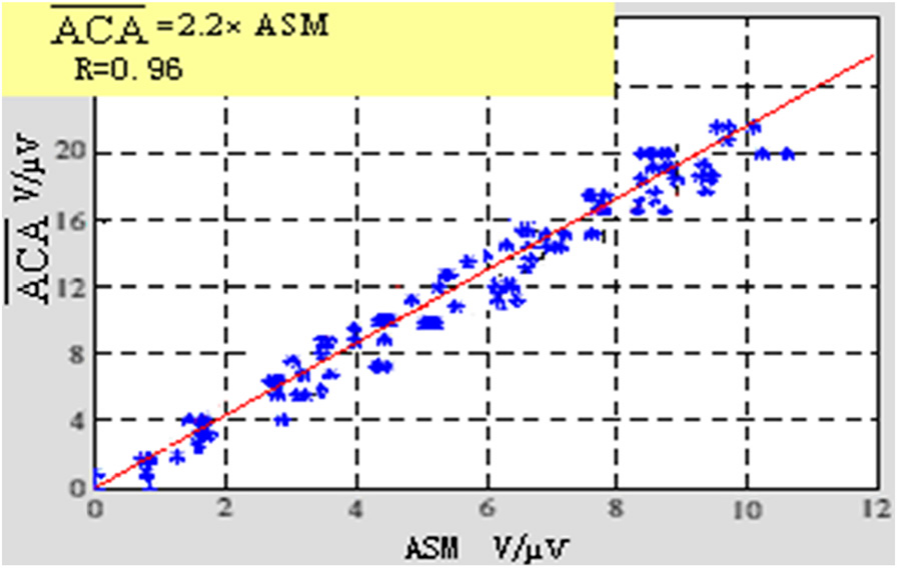
Measured TWA amplitude of CA vs. SM.

And the $A\overset{―}{C}A$ vs. ASM for the clinical data have the correlation coefficients of 0.96, which was shown as Figure 2.

For the patients of number 31, 41, 45, and e0121 in Table 4, the TWA presence frequency in measured ECG segments was above 70%. Consider that TWA is regarded as a marker for sudden cardiac death. These patients should be monitored in particular.

## Discussion

The definitions of T-wave onset and offset are not consistent in different literatures, and T wave is easily affected by the interference due to its lower frequency and morphological diversity. An empirical value method is used for extracting T wave in this paper [18]. The empirical value method may induce erroneous judgement in cases of RT interval variability, but it can avoid the difficulties to determine the onset and offset of T wave, and significantly reduce T wave detection calculation. For this paper T wave energy is required to be extracted, while the T wave energy is mainly concentrated in the middle part of the T wave, and its energy near T-wave onset and offset is very small, so the possibility of erroneous judgment caused by RT interval variability is reasonable small. Two segments of ECG data from the experimental signal were randomly extracted, which are used in the simulation test. The RT intervals are prolongation of 10 ms and shorten 10 ms, respectively, we found that the T wave energy extracted changes were less than 1%, and the Wilcoxon rank sum test results is no change, which showed that using empirical value method to extract T wave is proper.

The simulated ECG is obtained as a K-fold repetition of a single beat extracted from a real ECG. A perturbation, which waveform is generated by the Gaussian function, is added every other T-wave in this artificial ECG to simulate a TWA. And the amplitude of the added signal can be controlled. Four different noise sources from Gaussian white noise and physiological noise have been considered. The artificial ECG has the maximum approximation to clinical ECG, which guarantees the experimental results with the credibility. The ECG simulation scheme can be considered for performance evaluation of other TWA detection algorithms.

## Conclusions

TWA study has the important research value. An extensive scientific and clinical literature points to a fundamental link between TWA and susceptibility to life-threatening ventricular arrhythmias. Although some advances have been made in the TWA detection, it remains troubling to test TWA in the ambulatory ECG signals.

In this paper using the nonparametric test method combining with the CWT and correlation technique, a novel TWA detector algorithm was presented. The algorithm was validated using simulated ECG signals with artificial TWA of various amplitudes and noise levels. And for the clinical data test, SM was involved to detect and measure the same data with the algorithm, and their results have the greatly high correlation. TWAs were not only correctly detected qualitatively in frequency domain by energy value of T waves, but the alternans frequency and amplitude in temporal domain were measured by calculating *ACI*_*i*_ and $A\overset{―}{C}A$.

## Abbreviations

TWA: T-wave alternans; SCD: Sudden cardiac death; ICD: Internal cardioverter-defibrillator; SM: Spectral method; CWT: Continuous wavelet transform; ECG: Electrocardiogram; CA: Combined algorithm; ACI: Alternans correlation index; ACA: TWA amplitude.

## Competing interests

The authors declare that they have no competing interests.

## Authors’ contributions

XW and YZ carried out the study design and drafted the manuscript, KY collected patients’ data, DL participated in the design of the study. All authors read and approved the final manuscript.
